# Fifty years of a public cassava breeding program: evolution of breeding objectives, methods, and decision-making processes

**DOI:** 10.1007/s00122-021-03852-9

**Published:** 2021-06-04

**Authors:** Hernán Ceballos, Clair Hershey, Carlos Iglesias, Xiaofei Zhang

**Affiliations:** 1 International Center for Tropical Agriculture (CIAT), Cali, USA; 2 Alliance of Bioversity International and the International Center for Tropical Agriculture (CIAT), Alliance, Rome Italy; 3 Private consultant, Flinton, PA USA; 4 North Carolina State University, Raleigh, USA

## Abstract

This paper reviews and analyzes key features from cassava breeding at the International Center for Tropical Agriculture (CIAT) over 50 years and draws lessons for public breeding efforts broadly. The breeding team, jointly with national program partners and the private processing sector, defined breeding objectives and guiding business plans. These have evolved through the decades and currently focus on four global product profiles. The recurrent selection method also evolved and included innovations such as estimation of phenotypic breeding values, increasing the number of locations in the first stage of agronomic evaluations, gradual reduction of the duration of breeding cycles (including rapid cycling for high-heritability traits), the development of protocols for the induction of flowering, and the introduction of genome-wide predictions. The impact of cassava breeding depends significantly on the type of target markets. When roots are used for large processing facilities for starch, animal feeding or ethanol production (such as in SE Asia), the adoption of improved varieties is nearly universal and productivity at the regional scale increases significantly. When markets and relevant infrastructure are weak or considerable proportion of the production goes for local artisanal processing and on-farm consumption, the impact has been lower. The potential of novel breeding tools needs to be properly assessed for the most effective allocation of resources. Finally, a brief summary of challenges and opportunities for the future of cassava breeding is presented. The paper describes multiple ways that public and private sector breeding programs can learn from each other to optimize success.

## Introduction

This paper reviews and analyzes key features and results from cassava breeding at the International Center for Tropical Agriculture (CIAT) over 50 years and draws lessons for public breeding efforts broadly (Byerlee and Lynam 2020). The review discusses the evolution of objectives, strategies, methodologies, progress and outcomes; lessons learned; and perspectives on how to adapt and build for the future. It provides insights into the relevance of organizational structure and decision-making processes, financial procurement and resource allocation, and value chains targeted.

The authors are former and current cassava breeders at CIAT, and span 40 of the center’s 50 years of history. The CIAT story is only one part of complex and interdependent global efforts committed to improving livelihoods through better cassava varieties. Partnerships are at the core of the cassava breeding program *modus operandi*, as in the CGIAR centers in general. One of the most significant changes over the past 50 years has been the extraordinary growth in institutions (and private companies to a lesser extent) working on cassava and providing direct or indirect support to cassava breeding. We refer specifically to only a few of them, but acknowledge that any mention of CIAT learning and achievements is the result of many collaborating people and institutions, particularly with IITA (International Institute of Tropical Agriculture, Nigeria).

Systematic cassava breeding began with the creation of CIAT and IITA along with national programs (such as those in India, Thailand and Brazil) by 1970. National programs in Africa and other Asian countries were created a few years later. The first varieties resulting from international programs began to emerge after 1985.

## Institutional context

### Origins

In 2017, CIAT celebrated its 50th anniversary. Cassava research has been a core part of the center’s portfolio since just a few years after its founding. Development of new varieties and training have been central activities, in support of national agricultural research systems (NARS). Germplasm was used directly as varieties for grower and end-user benefits, or as parents in NARS breeding efforts. There are several overviews of CIAT history, most notably Lynam and Byerlee (2017). Hershey (1994) provided an overall Cassava Program history during its first 25 years and Kawano (2003) described key results of breeding over 30 years. Malik et al. (2020) summarized the results of 50 years of cassava breeding and agronomy focused on SE Asia. The most granular treatment can be found in CIAT Annual Reports and numerous conference proceedings which detail breeding strategy, activities and results (see search options in https://cgspace.cgiar.org). Many dozens of peer-reviewed articles are published from results of CIAT’s breeding research.

### Evolution of structure, funding and decision-making

Table 1 places CIAT cassava breeding in four broadly defined *eras* and describes key characteristics of each—with reference to structure, funding, objectives, and technological advances. We refer to elements of Table 1 throughout the paper, especially to place specific topics in the broader institutional context.

**Table 1:** Generalized descriptions of three broad eras in cassava breeding at CIAT

DESCRIPTION	Era 1: Comprehensive program	Era 2: Transition to project-based	Era 3: Donor-driven goals	Future expectations
Approx. time frame	1970s and 80 s	1990s and 2000s	2010s to present	Present to 2030
Team composition & support	Full interdisciplinary cassava research team	Core research team with disciplinary support across CIAT	Small core research team relying on synergies with CGIAR system-wide programs	
Program management	Comprehensive long-term program-based	Program-to-project transition	Project-based	Project-based, with One-CGIAR coordination
Funding	Generous long-term core funding	Transition from core to project funding	Mainly short-term projects, including some from private sector	Combination of short-term projects and long-term support from One-CGIAR
Genebank management	By breeding program	Field: by Cassava Program In vitro: by Genetic Resources Unit	In vitro and cryo-conservation; by Genetic Resources Program and One-CGIAR genebank platform	
Drivers of objective-setting	Farmers’ needs to improve yields. Income and nutrition stability	Market needs for quality and low-cost food and feed	Traits for sector-specific demands Human nutrition and social equity	Genetic gain in farmers’ fields for the defined market segments
Key strategies	Partnership building Broad edapho-climatic zone adaptation Yield, DMC and pest resistance advances	Broadscale varietal release Target market refinements Systematic inbreeding Assessment of breeding value of progenitors First molecular map (1997) Research for doubled-haploids Genetic transformation	Broadscale economic impact from new varieties Trait discovery and deployment Genomic prediction implemented in cassava Gene editing	Multidisciplinary determination of market segments and product profiles Breeding program modernization and continuous improvement Genomics-assisted breeding Reciprocal recurrent selection
Key outputs/ achievements	Germplasm collection & evaluation Strengthened national programs Full-fledged recurrent selection program established Populations developed based on edapho-climatic zone adaptation Models for high yield under ideal conditions Advances in pest and disease resistance, yield, DMC	Extensive adoption of new varieties in Asia Advances in understanding quantitative genetics and application to breeding Participatory breeding methodologies Extensive germplasm exchange from LAC to Africa	Key quality traits evaluated in genebank *Waxy* gene discovered and developed in released varieties Rapid cycling breeding for biofortified cassava High-throughput protocols based on NIRs and ground penetrating radar	Improved populations and varieties for defined market segments with > 1.5% genetic gain per year Game-changing traits identified from the genebank and integrated into breeding pipelines Enhanced genetic gain by exploring heterosis and implementing hybrid breeding

Cassava breeding efforts at CIAT were initially integrated within a broadly multidisciplinary research team, a model that inspired replication in many national research programs. This model allowed for an optimized allocation of resources based on a productive interaction among supporting areas of research. There was a common goal and each discipline contributed to it. The breeders drew on research and advice from entomologists, phytopathologists, agronomists, physiologists, economists and others to define strategy, breeding objectives and methods.

To promote and strengthen interactions within each discipline across different crops, CIAT changed its structure from *programs* to *projects* by the mid-1990s. In practice, however, what had been an integrated and coordinated effort toward common goals turned into an atomized, uncoordinated and often competing set of activities. This change took place as financial resources to the CGIAR were increasingly allocated to support restricted objectives, often fitting narrow priorities of donor institutions. The ultimate effect of these changes was a negative impact on the efficacy of cassava breeding. For example, whereas initially entomology research within the Cassava Program contributed to breeding efforts, in later years the entomology project competed for resources (to some extent) with cassava breeding. The administrative divisions that the *project* structure created were a burden that the *program* structure did not have. In spite of these *silos*, collaboration between different projects was still possible, albeit more difficult.

Simultaneously with the shift to a project structure, donors drove cassava research to become more focused on *tools*, i.e., developing cutting-edge technologies, rather than an outcome-driven agenda where the most appropriate methods and tools are selected to solve priority problems. Marker-assisted selection (MAS), for example, should not be an end in itself, but a tool to make cassava breeding toward specific goals more efficient.

In 2010, as the culmination of a reform and reorganization of the CGIAR system, root, tuber and banana crops (RTBs) were combined into a coordinated, integrated system-wide program in order to take advantage of the synergies among these vegetatively propagated starchy crops. This has had major impact on cassava breeding, allowing greater access to human resources and technologies applied across crops and across research centers. The benefits have been especially notable in upstream technologies, where cost-sharing and efficiencies of scale are critical, and in the social sciences input. The CGIAR system is currently implementing a “*reformulation of its partnerships, knowledge, assets, and global presence, aiming for greater integration and impact in the face of the interdependent challenges facing today’s world*” (https://www.cgiar.org/food-security-impact/one-cgiar/).

Cassava breeding has been almost exclusively carried out in the public sector, but at the same time has learned and benefitted from private sector experience, expertise and resources. Major distinctions between private and public breeding programs are: the way financial resources are procured; the decision-making processes on resource allocation; and, more importantly, that non-profitable markets are often targeted by public programs. Public research institutions are more likely to have organizational structures that are not as flexible and amenable to foster synergies as compared to the private sector. Pingali and Kelley pointed out in 2007 an important trend within the CGIAR system: the relatively rapid rise of restricted funding. In the early years, most contributions were unrestricted, but by 1990 this figure had fallen to 74% and by 2006 to 36%. The trend has continued further and threatens the integrity of the system´s research agenda. Stable and predictable funding is especially crucial for long-term research like plant breeding. As stated by these authors, “*Disproportionate levels of restricted funding could move the CGIAR emphasis away from the generation of international public goods toward the adaptive and development end of the research spectrum. Piecemeal project funding could over emphasize short term outputs at the cost of reduced Center emphasis on long term strategic research. There is an overwhelming consensus now that the growing share of restricted funding is distorting research priorities, increasing transaction costs, and reducing the efficient use of resources*.”

Critical integration and programmatic planning, which contributed to the excellent progress achieved, were unfortunately lost. It is increasingly difficult to obtain resources to support the whole breeding structure, with a major shift toward short-term investment in novel technologies. Cassava breeding clearly benefits by having the flexibility and the resources to invest in high risk/high potential technologies; however, innovation should not be an end in itself. For sustained progress, there needs to be a practical balance between innovation and actual impact, as the best way to define the areas that benefit from financial support.

## **E**stablishment and management of the germplasm collection

Successful breeding relies fundamentally on suitable genetic diversity. CIAT initiated a series of germplasm collection expeditions as its first activity in the new cassava improvement efforts. By 1970, a large international collection from the center of origin of the crop, and from diverse ecosystems, had been established. In the five decades since then, CIAT has approximately doubled the number of landrace varieties with small collections and continuing introductions from partners. Today, 6155 accessions from 28 countries, several hundred elite breeding lines, and about 30 wild species of *Manihot* are conserved in the collection, using in vitro techniques. The collection has been widely used around the world. About 6500 shipments of accessions from the collection had been sent to 84 countries by the year 2019 (https://ciat.cgiar.org/what-we-do/crop-conservation-and-use/cassava-diversity/). Hershey (2010) reviewed cassava germplasm management on a global basis.

Understanding existing diversity in the germplasm collection received high priority throughout the history of the cassava breeding program. The CIAT Genetic Resources Program maintains a database including passport data, characterization and some agronomic data (CIAT cassava genebank database). All genebank evaluations (along with CIAT and IITA breeding program results, and more) are publicly available on the Cassavabase database managed by the Boyce Thompson Institute (https://btiscience.org/our-research/enabling-technologies/cassavabase-project/).

Initial priority (Kawano 2003) was given to evaluating the collection for harvest index, fresh root yield (FRY), and root dry matter content (DMC). This was followed by evaluation for pest and disease resistance and adaptation to multiple abiotic stresses. Although a wide range of useful variability has been identified in the collection (e.g., resistance to cassava green mites, whiteflies, bacterial blight, immunity to cassava brown streak virus (CBSD), waxy starch, etc.) there is still a need for a thorough screening to uncover additional value-added traits.

## Breeding objectives

### Drivers and rationale for objective-setting

CIAT has evolved through several overlapping phases of defining the drivers of objective-setting for cassava breeding, each building on new knowledge and a changing client landscape (Table 1). We broadly describe these drivers in terms of: farmers’ needs for high productivity varieties; production system adaptation, sustainability, eco-efficiency and adaptation to climate change; meeting market needs for quality and sector-specific traits; and human health and social equity concerns. Globally, there are four clearly defined cassava product profiles: (*i)* biofortified cassava with high pro-vitamin A for nutrition enhancement; (*ii)* good cooking quality cassava (fresh or processed) for human consumption; (iii) high dry matter yield cassava for industrial use and animal feed; and (*iv)* special cassava with low amylose for food industry (Ceballos et al. 2007; 2011; 2013; Tran et al. 2021). Most cassava varieties released around the world would fit one (or two) of these product profiles. This section describes the dynamic approach assumed by CIAT in the definition of breeding objectives through time.

Cobb and co-workers correctly highlighted in 2019 the importance of properly defining breeding objectives. They also concluded that, because of the lack of business development units or marketing departments, “*public-sector breeding typically takes at best an academic approach or at worst a speculative approach to determining trait targets and breeding strategies*.” This section provides evidence that CIAT and partner breeding programs, especially in SE Asia, have had unquestionable impact and have been successful by any measurable approach because of a sensible definition of the breeding objectives. CIAT’s experience suggests that the success of a cassava breeding program does not depend on the presence or absence of business development units as part of the program itself (as suggested by Cobb et al. 2019), but rather on intimate knowledge of their target value chains and their operational efficiency, and the nature of these value chains.

#### Improved farmer income and food security

The CGIAR breeding programs were initially broadly driven by a need to curb food shortages and raise farmer incomes in regions of Asia, Africa and Latin America. This placed yield potential front and center as a breeding objective, and cassava was no different. Physiologists defined an ideotype for high yield, highlighting leaf area index as a critical determinant of yield potential under ideal conditions (Cock et al. 1979). Based on experiences from other crops, and with early experimental evidence in cassava, breeders focused strongly on harvest index as a selection criterion in the first years of breeding (Kawano 2003).

#### Adaptation, stability and sustainability in diverse production systems

As evaluation of germplasm expanded to different environments in the mid- to late 1970s, the CIAT program gained extensive new insights from soil science, entomology, pathology, physiology and agronomy. This new information allowed better understanding of the real-world conditions that farmers were facing, or were likely to face in the future, with the adoption of improved production practices and expansion into new production areas. With this growing knowledge base about the multiple biotic and abiotic factors impacting cassava growth and development, the cassava team took a closer look at best ways to stratify breeding objectives and develop populations for optimum genetic gain.

Our strategy aimed at varieties that responded well to low or moderate inputs (as opposed to adaptation in very low fertility or poorly managed conditions). More recently, climate change has increasingly influenced breeding objectives. Jarvis et al. (2012) suggested that changes in pest and pathogen populations would drive breeding objectives in cassava more than the direct effects of higher temperatures or drought.

#### Targeted value chains and markets

CIAT recognized early on the relevance that different value chains have in defining breeding objectives. Cassava farmers in SE Asia sell their harvests for the industrial production of starch, dried root chips for animal feeding and, more recently, ethanol. In these circumstances, defining a cassava ideotype is relatively simple: erect plant architecture, quick and uniform sprouting after planting, adequate harvest index, and maximized dry root yield (DRY) (Kawano and Cock 2005). Root quality traits such as cyanogenic potential are largely irrelevant for these markets.

Boiled cassava and different ethnic food products such as gari, fufu and batons require specific root quality traits (e.g., low cyanogenic potential, mealiness, poundability and/or swelling power). The artisanal processing setups at the farm or village level (common for ethnic products) offer a sharp contrast with the large facilities for industrial products (Johnson et al. 2003; Scott 2021). The drivers and the establishment of breeding objectives for varieties for human consumption are generally more complex than those for industrial uses for several reasons: consumer preferences can be quite specific and vary significantly even within a given region (and sometimes between men and women); the scarcity of high-throughput phenotyping tools for preference-related attributes; little understanding of their biochemical and genetic basis (except for cyanogenic potential); trait preferences as expressed by farmers and consumers are complex and cannot easily be converted into quantifiable indicators for breeding objectives (Valle 2021) and strong GxE interactions. More importantly, on-farm consumption and local traditional markets tend to be resistant to change.

In the case of Latin America and the Caribbean (LAC), farmers would often sell the roots to the fresh market when the price was favorable, or otherwise, to processing facilities (which generally pay less compared to the fresh market, but can absorb much larger volumes). At the inception of the Cassava Program, most of the varieties grown by farmers in Colombia could be called *dual-purpose*. Accordingly, the breeding program initially attempted developing varieties that could serve two vastly different markets. During the 1980s, however, it became evident that the dual-purpose breeding objective was not feasible: genotypes with outstanding dry root yield (ideal for the industry) would be rejected, for example, because of high cyanogenic potential. As a result, two distinctive product profiles were defined: direct human consumption and industrial cassava.

A case study for Asia illustrates the impact of markets on success in breeding. Table 2 distinguishes Asian countries where cassava is mostly processed into starch, ethanol or dried chips from those where cassava is often consumed directly or processed into ethnic products. More than 95% of cassava grown in the first group involved improved varieties. On the other hand, adoption of improved varieties where cassava is consumed directly is around 55%. Table 2 illustrates the way markets drive cassava breeders’ objectives and influence adoption of improved varieties. When most of the roots are sold for processing at large facilities connected to international markets, the ideotype is simple and clearly attained (Gracen et al. 2018; Kawano 2003; Kittipadakul et al. 2017); adoption and replacement of improved varieties are remarkably high and dynamic; and markets provide effective feedback to breeders if the targets are not set properly (or if there are arising new constraints or opportunities (as illustrated later on the subsection on novel starches).

**Table 2:** Adoption of improved cassava varieties in different Asian countries (extracted from Labarta et al. 2017) and production parameters from years 2014–2018 (FAOSTAT 2020)

Country	Adoption of improved varieties (% of total area planted)	Area planted to cassava (000 ha)	Average yield (t/ha)
Countries where cassava is not commonly used for direct consumption			
Cambodia	100	287	26.9
Vietnam	95	547	19.0
Thailand	99	1377	22.7
Across 3 countries	98.5 (average)	2210 (total)	22.9 (average)
Countries where cassava is mainly consumed and/or processed directly by farmers			
India	69	214	23.9
Indonesia	47	849	23.7
Philippines	45	226	12.0
Across 3 countries	54.5 (average)	1289 (total)	19.9 (average)

#### Public health and social equity

One of the central goals increasingly adopted across the CGIAR system is that breeders should address public health and social equity in developing product profiles. This can be immensely complex and difficult and is currently a matter of intensive research, including for cassava, and especially for gender issues (Mateo and Ortiz 2013; Forsythe et al. 2021). These concerns (not considered in Cobb et al. 2019) create an important distinction between the main driver of profit of private sector, and the wider scope of public breeding programs (https://www.corteva.com/who-we-are/outlook/securing-food-through-sorghum.html).

### Examples of meeting needs through specific objectives and breeding solutions

By following the collaborative CGIAR-NARS model, the CIAT Cassava Program has successfully defined and adjusted its breeding objectives through time. Successes and failures contributed along the way, as often occurs in any scientific endeavor. This section describes the way breeding objectives were initially defined, their evolution and perspectives for the future.

#### Adaptation and performance in the face of biotic and abiotic constraints

One of the first tasks of the breeder is often to assure that a population or a candidate variety is adapted in its target environment. In order to achieve a rational first-level stratification of breeding objectives, CIAT defined seven *edapho-climatic zones* (ECZs) (Hershey 1994). Each ECZ is characterized by a set of soil and climate characteristics, along with common pests and diseases, and provides the foundation for determining the target mega-environments of cassava production. Colombia has the good fortune to have a very diverse geography and climate, along with a wide range of the pests and diseases affecting cassava. This allowed CIAT to define testing sites for all the ECZs within Colombia (except the subtropics) and to identify homologs globally (Hyman 2020).

#### Improved nutritional quality

**Fig. 1:**
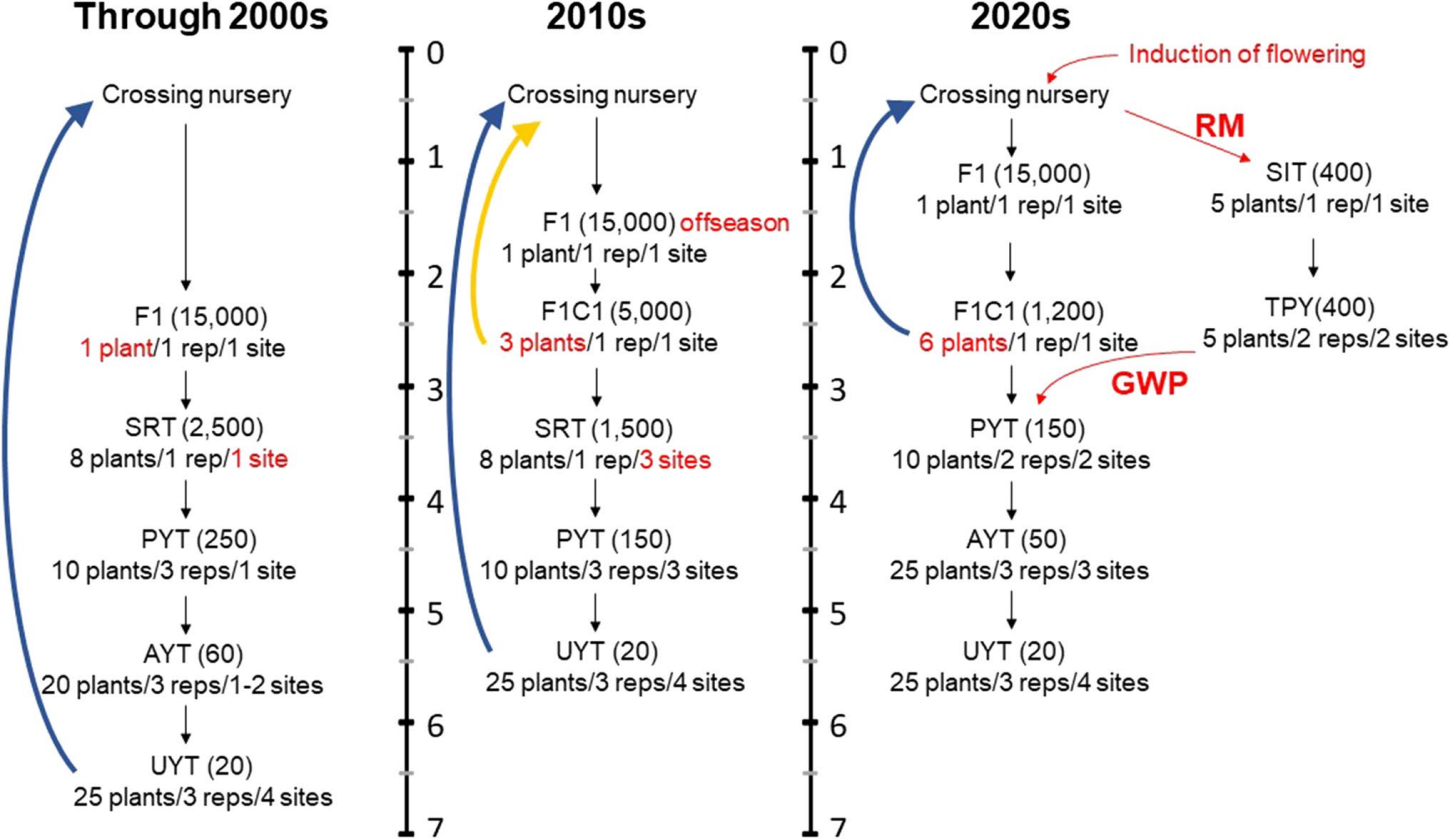
The evolution of breeding schemes at CIAT. F1: seedling nursery; F1C1: cloned seedling nursery; SRT: single row trial; PYT, AYT and UYT: preliminary, advanced and uniform yield trials, respectively; GWP: genome-wide prediction; SIT: seed increase trial; TPY: training population yield trials; RM: rapid multiplication. Upward arrows indicate the duration of each breeding cycle (the orange arrow is for the rapid-cycling for high-carotenoids breeding)

The innovative approach to improve nutritional quality in different crops (biofortification) arose entirely within public breeding programs (Bouis and Saltzman 2017) and included cassava since its inception (Bouis et al. 2017). These efforts were duly acknowledged by awarding the 2016 World Food Prize in recognition of the work developing orange-fleshed sweet potato. By the late 1990s, initial screening was conducted to assess variation in nutritional quality within the cassava germplasm collection (Iglesias et al. 1997). The program eventually focused just on pro-vitamin A carotenoids (Chávez et al. 2005). Significant progress was achieved after ten years of breeding (Ceballos et al. 2013), based initially on a rapid-cycling approach specifically designed to take advantage of the high-heritability of carotenoids content in cassava (Fig. 1). To achieve and sustain genetic progress, the program developed appropriate sampling strategies (Ortiz et al. 2011) and created an efficient high-throughput phenotyping approach based on near-infrared spectroscopy (Belalcazar et al. 2016; Sánchez et al. 2014). Public breeding programs are in a unique position to set visionary breeding objectives that are beneficial to society, even though these benefits are not initially evident to consumers. Biofortification is now a widespread breeding objective worldwide, including within the private sector.

#### Discovery and exploitation of novel starch quality traits

Cassava is the second most important source of starch worldwide, after maize. The starch sector requested in 2000 a waxy starch (amylose-free) cassava variety and provided financial support to screen the germplasm collection in search of the trait. Eventually, a waxy starch mutation was identified in 2006 (Ceballos et al. 2007). A second mutation (small-granule starch) was discovered around the same time, but in a population resulting from gamma-ray mutagenesis of true seeds (Ceballos et al. 2008).

In the years following the discovery of these mutations, intensive work was conducted to assess their functional properties (Rolland-Sabaté et al. 2012; 2013; Sánchez et al. 2010), which expanded the markets for cassava-based products. More importantly, research agreements were signed to develop waxy commercial varieties for SE Asia and South America with close involvement and the financial support of starch companies. The first generation waxy-starch cassava varieties were released in Thailand in 2013 (Rojanaridpiched et al. 2020; Thai Tapioca Development Institute, 2017), and the first commercial processing of waxy cassava starch in Colombia took place in 2019. Starch quality research has reflected the dynamism behind defining product profiles in close interaction with relevant market players.

#### High and stable dry matter content (DMC)

High and stable DMC is a trait highly appreciated by farmers and processors alike. The quality of cassava roots (and their selling price) fluctuates with the age of the plant and the environmental conditions prior to harvest. Farmers typically harvest cassava near the end of the dry season because DMC normally reaches a maximum and the duration of the storage period of stems for next season’s planting is shorter. Erratic rainfall is one of the most common consequences of climate change and has been observed in the sub-humid environment of Colombia.

Cassava germplasm with high DMC has already been developed and deployed. It is desirable, however, to add the requirement that these high DMC levels are relatively stable (Ceballos et al. 2011). However, screening for stable DMC is not a trivial endeavor and is highly affected by GxE interactions (Sagrilo et al. 2008). Management complicates matters further (GxExM). Trials were grown for up to 18 months, disrupting the normal operations and creating logistic challenges. For several years, CIAT selected genotypes with high DMC at normal harvesting age (10–12 MAP) and combined with capacity to maintain acceptable levels after the arrival of the rains (15–18 MAP). Extended harvest (e.g., at 18 MAP) is a common practice in subtropical regions of South America, particularly in Southern Brazil (Sagrilo et al. 2008). A set of eight experimental clones selected for high and stable DMC is currently under evaluation for their release as commercial varieties in Colombia (Lenis et al. 2018). Ideal genotypes are those with a competitive performance (in terms of FRY and DMC) in normal harvests and show a minimum reduction in DMC in extended harvests, after the arrival of the rains. In the latter case, unstable dry matter is mainly determined by the mobilization of starch from the roots at or after stressful conditions.

#### Understanding and dissecting quality traits for human consumption

The adoption of improved cassava varieties for boiling and ethnic uses is limited. Several factors contribute to this situation, including the fact that released varieties often fail to meet the required quality traits (Eriksson et al. 2018). One major bottleneck to breeding is the limited information regarding the genetic and biochemical basis determining root quality traits for different end uses, and suitable high-throughput phenotyping protocols. In contrast, potato breeders have made significant advances in understanding tuber quality traits and developing phenotyping tools that facilitate selection (Dufour et al. 2021; Morris and Taylor 2019; Sharma et al. 2020). The project *RTBfoods: Breeding Roots, Tubers and Banana products for end-user preferences* (https://rtbfoods.cirad.fr) is an ambitious initiative to elucidate basic information regarding root quality traits. Several research articles regarding root quality traits and post-harvest processing have recently been published (Alamu et al. 2021; Bechoff et al. 2018; Dufour et al. 2021; Escobar et al. 2021; Goddard et al. 2015; Iragaba et al. 2021; Luna et al. 2021; Ndjouenkeu et al. 2021; Teeken et al. 2018, 2021; Thiele et al. 2021; Tran et al. 2021). The availability of this critical knowledge and reliable high-throughput phenotyping protocols will improve the efficiency of breeding for root quality traits.

#### Preparing cassava for the CO_2_ levels of 2050

Cassava is photosynthetically highly efficient among C_3_ species (El-Sharkawy et al. 1992; El-Sharkawy 2014; 2016). The crop competes with maize in the starch, animal feed and ethanol value chains. Increased concentration of atmospheric CO_2_ is one of the causes of global warming and it is expected to continue rising through the year 2050. This rise has beneficial effect on photosynthetic efficiency and productivity in cassava (Rosenthal et al. 2012; Ruiz-Vera et al. 2020), and may reduce the physiological advantage of C4 crops, such as maize, over cassava. Breeders should exploit the opportunity to select and breed for positive responses to rising CO_2_ levels and minimize detrimental effects (if any). Crops can be grown in the field under set CO_2_ levels using free-air concentration enhancement (FACE) rings. So far, however, these trials have only been conducted in temperate regions of North America and would benefit from replication in the tropics where cassava is grown.

## **O**ptimizing breeding strategies, methods and tools

### Filling gaps for basic information

Public sector breeding often covers crops that offer lower opportunities for profit by breeding enterprises. The absence of commercial opportunities is closely linked with a lower level of investments for research. In the case of cassava, there was a remarkable lack of basic information at the time CIAT and IITA were created. In spite of huge advances, there remains a significant knowledge gap relative to other major crops. As one important example, critical information regarding the flowering biology of cassava (receptivity of stigmas, pollen tube growth, embryo development) has only been addressed in the past three years (Lentini et al. 2020a; 2020b; 2020c; Pineda et al. 2020a; 2020b; Ramos-Abril et al., 2019). Unfortunately, this lack of relevant basic information resulted in a serious bottleneck for the development of new technologies such as protocols for the production of doubled-haploids. Likewise, limited information about the genetic and biochemical determination of root quality traits has had detrimental effect on the development of varieties targeting human consumption of cassava.

### Optimizing phenotypic recurrent selection

Many breeding programs have used the same basic conventional cassava breeding method for decades, with variations introduced as new information became available (Ceballos et al. 2012, 2017; Gonçalves Fukuda et al. 2002; Jennings and Iglesias 2002; Kawano and Cock 2005; Hershey 2020a, b). The first step involves the production of full- or half-sib seed in crossing nurseries. A major challenge of seed production in cassava is the association between quantity of seed produced and plant growth habit. Early, frequent and abundant flowering is closely associated with a highly branched plant architecture, which is undesirable (Ceballos et al. 2015). Farmers prefer an erect architecture, which results from late (or absent) flowering. Producing seeds from progenitors with erect plant architecture is, therefore, a challenging task. Only recently, progress to accelerate the production of botanical seed has been achieved through different strategies (Hyde et al. 2019; Pineda et al. 2020a; 2020b; Silva Souza et al. 2018). Cassava progenitors are heterozygous and even full-sib families are genetically very diverse. Each F_1_ seedling is genetically distinct and several years are required to produce enough stem cuttings for multi-location testing (the multiplication rate in cassava can vary widely, but is about 1:10 on average).

Figure 1 depicts the evolution of breeding schemes. Starting in the 2010s, the seedling nursery (F1) was planted in the off season and grown for only six months. The F1C1 stage with three plants per genotype was added as the source of planting materials for the following single row trial (SRT), which could then be planted in three locations (red text). The following stages are the preliminary (PYT), advanced (AYT) and uniform (UYT) yield trials with increasing number of plants per plot, but AYT was no longer grown in the 2010s**. **Selected genotypes were incorporated to the crossing nurseries (blue upward arrows). Rapid-cycling (Ceballos et al. 2013) was implemented for high-heritability traits such as carotenoids content (orange upward arrows).

The development and implementation of the flower-inducing technology shortened the duration of crossing nurseries in the 2020s (Fig. 1)**. **The F1 is grown for seven months, which allows F1C1s to have six plants per genotype and then PYT to grow in two locations. Selections for PYT will be made based on the total genetic value predicted using genome-wide prediction (GWP). The F1C1 clones with the best predicted breeding value will be cycled back to crossing nurseries as progenitors for the next cycle of improvement (black upward arrows). The GWP training population is selected from the breeding population based on the pedigree. Rapid multiplication (RM) is performed in green house to obtain five plantlets from each seedling. The plantlets are transplanted into the seed increase trial (SIT). The following growing cycle, the training population yield trials (TPY) are established at two locations for phenotyping. The SIT and F1C1 clones are genotyped for genome-wide prediction.

The traditional evaluation process has been extensively described (Jennings and Iglesias 2002; Ceballos et al. 2012; Hershey 2020a, b). Selection in the seedling nurseries (F1), which at CIAT involves ≈15 thousand genotypes per product profile, is only for general vigor and high-heritability traits such as resistance to specific pests and diseases and occasionally for carotenoids content or starch quality traits. In Africa, drastic selection takes place to eliminate genotypes susceptible to CMD. Selected seedling plants are cloned and single row trials (SRT), preliminary (PYT), advanced (AYT) and uniform (UYT) yield trials follow with a gradual reduction of genotypes evaluated while plot size, number of replications and locations involved increase.

Starting in the 2010s (Fig. 1), a modification of the breeding scheme was implemented to overcome logistical problems in breeding for high-carotenoids (Belalcazar et al. 2016), but the new experimental design was quickly implemented for every product profile, to improve accuracy and reliability. The F1 is germinated in August each year (six months earlier than in the traditional system). Seedlings are grown in the field for only six months. At this age, selection for key traits (vigor, plant architecture, roots with yellow parenchyma or waxy starch, reaction to thrips) can be made but only three stem cuttings can be taken. Each selected genotype is thus cloned and planted in a new stage (F1C1) at the normal planting time and grown for a year. This approach allows growing identical SRTs in three locations, overcoming a major weakness of the original system (Fig. 1). Genotype-by-environment (GxE) interactions limit the reliability of SRTs (Adjebeng-Danquah et al. 2017; Akinwale et al. 2011; Chipeta et al. 2017; Mtunguja et al. 2016; Peprah et al. 2016; Ssemakula, and Dixon 2007). Preliminary results of this innovation indicate that only 25–35% of clones are simultaneously selected in two of the three locations, highlighting the effect of GxE in these non-replicated trials. Changes implemented in the 2020s are described later.

### Exploiting phenotypic breeding value

Based on breeding theory, CIAT investigated the possibility to exploit breeding values for more effective progenitor selection. Single row trials (SRT) include several full- and half-sib families, each one represented by varying numbers of genotypes. It was common that a given progenitor was used as a parent in more than one family. Data were taken from both selected and rejected genotypes in the SRT, allowing for an approximation of the general combining ability (GCA) of each progenitor (Ceballos et al. 2004). A large data set involving more than 30,000 plots through 14 years of evaluation in the sub-humid environment tracked the performance of thousands of genotypes evaluated from SRT through uniform yield trials (UYT), and was later published (Ceballos et al. 2016a, b). In spite of promising early results, selecting progenitors based on GCA proved inefficient. Large within-family genetic variation relative to differences in breeding values (Ceballos et al. 2016a) and the relevance of non-additive genetic effects (Ceballos et al. 2015, 2017; Wolfe et al. 2016) are partially responsible for the limited impact that selecting for GCA had.

Another important factor is *phenotypic instability*. The phenotype of a given genotype in the SRT may not be the same four years later at the UYT (Joaqui et al. 2016). Breeding clonally propagated crops requires consideration of the impact of the overall quality of the planting material. Epigenetic factors, nutritional and physiological status, pathogens and beneficial endophytes (Bräutigam et al. 2013; Jansky and Spooner 2018) may affect the general performance of the same genotype through the sequential stages of evaluation depicted in Fig. 1. These factors may partially explain the poor correlations observed in the phenotypic evaluation of the same genotypes through these different stages of selection (Joaqui et al. 2016) and, to some extent, justify a lengthy evaluation process. Phenotypic instability complicates considerably the breeders´ equation described in Cobb et al. 2019.

### Genotyping and genomic prediction to accelerate genetic gain

The rapid improvement of DNA sequencing technologies has driven down the cost to less than one penny per megabase of DNA sequence in 2020 (www.genome.gov/). Sequencing-based marker technologies, in turn, are dramatically reducing the cost of genome-wide marker discovery. High density genome-wide markers, combined with statistical tools, have the potential to enhance conventional plant breeding, not only in identifying and validating trait markers for MAS and implementing genome prediction (Rabbi et al. 2020; Wolfe et al. 2017), but also in characterizing and managing genetic variance and performing optimum contribution selection when making crosses (Hallander and Waldmann 2009; Wang et al. 2016a, b). Collaborating with Beijing Genomics Institute, CIAT is using whole genome sequencing to discover genome-wide markers from cassava breeding populations. The high-density markers are used in association mapping and linkage mapping to identify major QTLs in breeding populations, and provide detailed information about the haplotypic diversity, to design diagnostic markers for MAS. Moreover, with the genotypic data, we are able to calculate the genomic best linear unbiased prediction (GBLUP) of breeding materials, and make more accurate selection in variety development than pedigree BLUP or replication mean.

The genotyped breeding populations also serve as the training populations for genomic prediction. Considering the determinant of genetic relationship on the prediction accuracy, we use a small subset of the breeding population, selected based on the pedigree, as the training population. The clones in the training population and the breeding population are siblings, which leads to high prediction accuracy. On the other hand, rather than genotyping the entire breeding population (~ 10,000 seedlings per product profile), we rely on the cheap seedling nursery to get rid of more than 80% clones of the breeding population, and only genotype the selected clones for genomic prediction. Moreover, the training population starts from seeds and we are able to share the seeds with collaborators, with a simple quarantine inspection process. The training population is evaluated in the collaborator’s environment, and the collaborator-specific genomic prediction models will be developed for progenitor selection from the breeding population at CIAT. We will use the progenitors to produce seeds as improved populations and share them with collaborators for variety development.

Genomic predictions, therefore, will facilitate the germplasm sharing and enhance CIAT’s capacity of improving populations for NARS. Now we are implementing this genomic prediction strategy with the breeding programs in Southeast Asia to develop cassava varieties with high and stable dry matter yield and CMD resistance. Compared with the current conventional breeding scheme, a genomic prediction-based breeding scheme will reduce duration of the selection cycle by at least two years (Fig. 1). The genomic prediction strategy is also implemented in the breeding pipelines for the introgression of CMD and CBSD resistance into the high pro-vitamin A pipeline and good cooking quality pipeline for Africa. To bring all breeding data together to inform breeding decisions, we fully use CassavaBase to manage phenotypic data, genotypic data and pedigree data (Fernandez-Pozo et al. 2015).

### Development and application of high-throughput phenotyping tools

CIAT and partners have contributed proactively to developing reliable and affordable phenotyping protocols to overcome bottlenecks in the breeding process. The use of near-infrared spectroscopy (NIRs) on fresh root samples greatly facilitated quantification of carotenoids contents (Sánchez et al. 2014; Belalcazar et al. 2016; Alamu et al. 2021). It is now widely used in different biofortification projects. The use of ground penetrating radar for the non-destructive assessment of root growth through time is another ambitious innovation in which CIAT actively participated (Delgado et al. 2017). This technology would be relevant for assessing early bulking, an elusive trait often demanded by farmers. Important progress for facilitated measurements of cooking time has recently been published (Tran et al. 2021), which will facilitate breeding for varieties consumed directly after boiling the roots. Aeroponics techniques are now refined and should be an option for rapid screening of early rooting characteristics (Selvaraj et al. 2019). Remote sensing via drones to analyze growth patterns is under development (Selvaraj et al. 2020). Combined with data from underground root development, this can provide valuable tools to integrate real-time whole-plant development with yield potential, and ultimately result in high-throughput phenotyping tools.

### Participatory approaches in priority setting and evaluation strategies

In response to the limited adoption of varieties for direct consumption in resource-limited farming conditions, the cassava breeding program, along with the socio-economics unit at CIAT, pioneered the development and application of farmer participatory approaches. Beginning in 1986, CIAT and Colombia's National Research Program worked collaboratively to develop, refine and apply a methodology for utilizing farmers' expertise and knowledge about their variety needs (Hernandez 1993; Hershey 2020c). Participatory approaches are likely to facilitate development of varieties that out-perform those developed through conventional methods when they are applied in low-yield cropping systems and in specific locations.

Participatory breeding has evolved over time. Mother–baby trials (Atlin et al. 2001; 2002; Ramisch 2012; Snapp 2002) combine larger trials done on-station or on-farm (mother trials), with smaller on-farm, farmer-managed trials of 1–3 technologies (baby trials). More recently, the TRICOT design (triadic comparisons of technology options) has been suggested and tested (Steinke et al. 2017; van Etten et al. 2019a; b; 2020), including in the NextGen cassava project (https://www.nextgencassava.org).

## **O**utputs and impact of cassava breeding

Crop genetic improvement has historically been the bread and butter of the CGIAR. Improved germplasm for use by national programs, either for direct release or as parent material, is a classic international public good with demonstrated large spillovers across regions and countries (Maredia and Byerlee 2000). There are several ways in which results from cassava breeding can be measured, ranging from the biological outputs (genetic gains), to adoption by farmers (economic impact). The training and education of future plant breeders are another important contribution and a primary role of the public plant breeder (Fowler 2005), although it is difficult to assign a quantitative value to this contribution. Equally difficult to quantify is the overall impact that cassava research at CIAT has had in consolidating the cassava breeding system, which includes NARS.

### Genetic gains

Estimations of the impact from breeding at the CGIAR centers, from the time of their creation, on the productivity of many crops have been published (Alston et al., 2020; Evenson and Gollin 2007; Pingali 2012; Pingali and Kelley 2007; Renkow and Byerlee 2010). For cassava, gains are very diverse across African countries, but there are several studies demonstrating significant increases in productivity and sizable returns on investment (Eriksson et al. 2018). The genetic gain per year in Africa has been estimated at 1.3% for FRY and 1.2% for dry root yield (Okechukwu and Dixon 2008). Similarly, genetic gains in Thailand were estimated to be 2.50 and 1.06% and per year for FRY and DMC, respectively (Kawano 2003). During the 1975–2015 period, annual estimated gains were 0.23 t/ha for FRY, 0.13% for starch content and 0.12 t/ha for root dry yield (Ceballos et al. 2020; Kittipadakul et al. 2017). Rapid-cycling for enhanced carotenoids content resulted in consistent annual increases of 2.06 ug/g for total carotenoids content (fresh weight basis) over an eight years period (12% per year gains). These remarkable gains could be achieved because of the high heritability for this trait (Ceballos et al. 2013).

### Economic impact

Johnson and co-workers in 2003 estimated economic benefits from the use of improved cassava varieties in 1998 alone at (million US$) 12.9 for LAC (7 countries), 335.7 for Africa (20 countries) and 99.5 for SE Asia (5 countries). When total investments in cassava breeding at CIAT are considered, the internal rate of return was 22%. The impact of breeding within the CGIAR has been achieved in spite of a sharp decline in share of its core funding (funding with considerable flexibility for internal institutional management) over time, falling from 25 to 16% between 1992 and 2005 (Pingali and Kelley 2007). Investments in cassava breeding at CIAT fell by 30% from 1990 to 1997 (Johnson et al. 2003).

In addition to this reduction in the overall investment in crop breeding, a considerable proportion of the investment was diverted to new emerging technologies (namely genetic transformation and molecular markers research). However, to date, there is no commercially grown genetically modified cassava. Twenty-five years after the publication of the first molecular map of cassava, there is no cost/benefit report of MAS. Outputs from conventional cassava breeding risk a slowdown as a result of competing investments in novel technologies that have not yet had any measurable impact in productivity. As stated by Cobb et al., (2019), breeding teams need to evaluate carefully the impact of any new technology and such evaluation is long overdue. Contrary to suggestions in the literature, there is no evidence that the rates of return to research within the CGIAR had declined over time (Pingali 2012).

### Sustainability and yield stability

Yield stability is important especially for poor farmers whose food security and livelihoods are vulnerable to chronic or fluctuating biotic and abiotic stresses. Recent evidence suggests that later generations of improved crop varieties have stabilized yields in comparison with those they replaced (Renkow and Byerlee 2010). In the case of cassava, resistance or tolerance to prevalent abiotic or biotic stresses is among the most relevant traits considered by the breeders. For example, in the late 1980s and early 1990s, a new strain of CMD (UgV/EACMV-Ug) emerged in East Africa. Losses to cassava production have been estimated at 140,000 tons annually, which is equivalent to USD 14 million (Legg and Thresh 2000). However, no major CMD epiphytotic has been reported in the last 30 years in Africa after the identification and deployment of new sources of resistance (Eriksson et al. 2018).

Public breeding programs in East Africa have now released varieties tolerant/resistant to CBSD, another threat to cassava production in Africa (Kawuki et al. 2016). Several accessions from CIAT’s germplasm collection were found to be immune to CBSD at the greenhouse level and are now undergoing field trials (Sheat et al. 2019). This is the first time such a strong type of resistance has been reported for CBSD. Incorporation of genes for resistance to CMD and CBSD into elite varieties of cassava in SE Asia and Africa will be the most effective method to limit damage from the viruses (pre-emptively for CBSD in W Africa and SE Asia). However, the lack of homozygous progenitors makes the simple introgression through back-crossing impossible (discussed further below).

Developing varieties tolerant to abiotic stresses (particularly drought) is another output from cassava breeding programs, particularly for sub-humid and semi-arid environments (Burns et al. 2010; Okogbenin et al. 2014). However, cassava as a species is quite drought tolerant, and the impact on yield stability of varieties selected specifically for additional tolerance has not been reported so far.

### Public health and social equity

Breeding programs generally aim to improve the income of users of new varieties, but also may impact their nutrition and health status through biofortification (Bouis et al. 2017). This is an area of impact not considered by Cobb and co-workers (2019). CGIAR breeding programs (including those for cassava at CIAT and IITA) made significant efforts to improve the nutritional quality of different crops. These efforts did not translate in yield gains, but they certainly resulted in the delivery of valuable public goods. Their impact, however, will only be measurable in vulnerable populations, several years after biofortified varieties have been released. Meenakshi and co-workers demonstrated in 2007 that biofortification can have a significant impact on the burden of micronutrient deficiencies in a large number of countries in Africa, Asia and Latin America. More importantly, it can do so in a highly cost‐effective manner.

## **C**hallenges and new opportunities

We conclude the overview of cassava breeding by suggesting some priorities for the future based on current or arising challenges, and the tools, technologies and institutional innovations that can contribute to optimized breeding.

### Understanding and expanding the germplasm base

Hershey (2010) described the future needs for new initiatives to expand and ensure optimized use of the germplasm base available to cassava breeders. While there is ample evidence that breeding has not generally been constrained by lack of diversity, for a very wide array of target traits, there remain regions of likely new and high diversity that have not been adequately collected. Many of the wild *Manihot* species are at risk of extinction because of land clearing for agriculture and are vulnerable to climate change. New germplasm collection is currently not on the radar for major donors, and a case needs to be made that will change this.

CIAT is currently in the process of genotyping its cassava collections using DarTseq (to be completed in 2021). This will be the first full, high-resolution genotyping of a complete genebank of a major crop, and will be an invaluable resource for understanding the crop’s evolution, defining gene pools, gene discovery and more. Attention should be paid to managing genotypic data and phenotypic data linked with the test environment information and making the data publicly available and easily accessible. The genotypic data and phenotypic data are linked with the plots and the meta-information of yield trials. The features in CassavaBase provide the reference for developing a database to manage genebank information.

### Promising arising tools and technologies

The identification of useful traits in CIAT´s germplasm collection (for example waxy starch and resistance to CBSD) illustrate the potential of trait discovery for the future of the crop. It is reasonable to assume that new traits could be discovered within the wealth of genetic diversity encompassed by the global germplasm collection. The huge progress in understanding the *Arabidopsis* genome and the comparison with the sequenced *M. esculenta* genome could yield valuable information about gene functions in cassava. An array of useful traits ranging from herbicide tolerance, starch functional properties, haploid inducers, etc., could be identified using *molecular sieving tools* such as Eco-TILLING (Comai et al. 2004; Kumar et al. 2017). In spite of the early evidence demonstrating its feasibility (Duitama et al. 2017), there has been no further follow-up by the biotechnology community.

Gene editing is a reality in many crops, including cassava (Gomez et al. 2019; Mehta et al. 2019). A key issue regarding this technology is the gene sequence to be targeted. The haploid inducer technology has been widely used in maize and other monocots (Kalinowska et al. 2019) and, through wide crosses, in potato (Rokka 2009). Orthologues of the genes responsible for inducing haploids in monocots have been recently identified in dicots (Zhong et al. 2020). There is ongoing research at CIAT targeting a sequence expected to induce haploids in cassava. This initiative illustrates what has been a productive collaboration between field breeding and biotechnology. The development of protocols for the induction of earlier flowering and seed production (Pineda et al. 2020a; 2020b) facilitates inbreeding through successive self-pollinations. However, the easiest and fastest way to produce inbred lines would be through the induction of haploidy through the haploid inducer technology or tissue culture approaches (Lentini et al. 2020a, 2020b; Perera et al. 2012).

### The challenges and opportunities of trait introgression

Trait introgression is a key process in plant breeding and is likely to become even more prevalent in the age of gene discovery and editing. Back-crossing is a widely used and highly effective breeding method for trait introgression (Xu and Crouch 2008). However, it cannot be implemented in the classical manner in cassava, because of the lack of inbred progenitors. The cassava breeding project at CIAT introgressed the single recessive waxy gene to commercial varieties in SE Asia and South America. The introgression necessarily requires multiple generations of recombination to reduce the impact of the less desired alleles from the source parent of the waxy mutation(s). The first varieties to be released in Thailand, for example, had a starch productivity equivalent to that of Rayong 1, a landrace variety formally released in 1975 (Ceballos et al. 2020): about 45 years of breeding progress were reversed by introgressing a single recessive trait. Excellence in cassava breeding urgently requires an efficient way to introgress useful traits into selected backgrounds.

The issue of trait introgression has long been considered by cassava breeders (Ceballos et al. 2015). The introgression of waxy starch targets a specific, high-value sector of the starch industry. More urgent and global is the need to introgress resistance to CMD in SE Asia after the recent introduction and rapid spread of the disease in that region (Wang et al. 2016a, b). Although the best source of resistance to the disease (CMD2) has been fully characterized and markers have been developed (Kuon et al. 2019; Wolfe et al. 2016), its introgression remains highly inefficient (Ceballos et al. 2020). Similarly, the introgression of CBSD resistance identified from South American landraces (Sheat et al. 2019) into African varieties would be unnecessarily cumbersome. The obvious response is that cassava breeding must shift toward the use of inbred progenitors, so that efficient backcross (and MAS) could be finally implemented. Excellence in cassava breeding relies on controlling the way alleles segregate, rather than just tracking them.

### Pre-breeding and population improvement as a precursor to hybrid development

Recurrent selection is an efficient way to improve populations by gradually and consistently shifting allelic frequencies in the desired direction. By definition, recurrent selection operates on additive effects (e.g., GCA) or genomic estimated breeding value (GEBV) in phenotypic recurrent and genomic prediction, respectively. In contrast, when the final product is a hybrid rather than a population, a relevant part of the genetic variability defining the performance is non-additive.

Optimized cassava breeding requires efficient exploitation of non-additive genetic effects (Ceballos et al. 2015, 2016a, 2017). As stated by Bernardo (2020): “*The genotypic value is that of the candidate itself, whereas the breeding value is reflected by the mean of the candidate’s progeny when it is mated with random individuals*.” Therefore, in cassava and other vegetatively propagated crops, the prediction of total genetic value should be used for the advancement of clones toward later stages and potential commercial release. Advancing clones based on breeding values (additivity) could miss those combinations that have the added dominance and epistatic boost that would make them excel.

In the case of cassava, pre-breeding work through genomic predictions, complemented by standard phenotypic evaluation and selection protocols to identify outstanding hybrids has been proposed (de Oliveira et al. 2012; Wolfe et al. 2017). A key assumption is (as it was in the maize pipelines analyzed by Good in 1990), that successive cycles of population improvement would increase the probability of identifying superior hybrids. A process diagram of the breeding strategy should allow for transparency and accountability, and helps breeding management teams develop key performance indicators that assess the value of activities in delivering genetic gains (Cobb et al. 2019). The cost/benefit comparison of alternative cassava breeding approaches should be documented to support decision-making and research design.

### Exploiting heterosis and hybrid breeding

Heterosis is a critical phenomenon not only for commercial hybrid and RTB crops (Batte et al. 2020; Gopal 2014; Gurmu et al. 2018; Mendoza and Haynes 1974; Musembi et al. 2015; Quero-García et al. 2009), including cassava (Ceballos et al. 2015), but also for self-pollinated ones (Lin et al. 2020). Additive genetic effects explain a large proportion of the variability for most traits, and most can be efficiently improved through modern genomic tools (de Oliveira et al. 2012; Okeke et al. 2017; Wolfe et al. 2016; 2017). Although non-additive effects account for a smaller proportion of the genetic variability, they are critical for the performance of outstanding hybrids (Melchinger et al. 2007).

Maize remains the reference crop regarding the exploitation of heterosis. Key elements for the success of maize breeding have been the identification of heterotic groups and the use of inbred progenitors (Mikel and Dudley 2006). However, the identification of heterotic patterns such as those found in temperate maize has so far proven to be difficult in cassava. Genetic distances, as in the case of many crops, are not a good predictor for specific combining ability in cassava (Ceballos et al. 2016b). There are ongoing efforts to identify heterotic patterns among IITA populations (Peter Kulakow, personal communication). Such research is critical for a systematic exploitation of heterosis through reciprocal recurrent selection schemes and/or development of fully or partially inbred lines.

Recent breakthrough developments in potato breeding imitate maize breeding (Hosaka and Sanetomo 2020; Jansky et al. 2014, 2016, 2018; Lindhout et al. 2011, 2017). The possibility of developing inbred progenitors has been fundamental for this quantum leap taken by private potato breeding companies. If public sector breeding programs are to take advantage of the lessons from the private sector (as suggested by Cobb et al. 2019), then future cassava breeding should explore options to shift to the use of fully or partially inbred lines. Partial progress in that direction has already been made, with promising results regarding flower induction (Pineda et al. 2020a, 2020b), important progress toward the development of a protocol for the production of doubled-haploids in cassava (Lentini et al. 2020a, 2020b, 2020c), and the ongoing efforts to edit the cassava genome for the expression of haploid induction.

### Reverse breeding and haplotype breeding value

Successful hybrids are the combined result of positive general and specific combining ability effects. For example, KU50 is an outstanding cassava hybrid clone widely grown for decades in several Asian countries (Gracen et al. 2018). In a way, KU50 equals the memorable cross between Mo17 and B73 in maize. The heterotic pattern in this maize hybrid has been fundamental for the hybrid maize industry (Mikel and Dudley 2006; Stuber 1994). Over the years, different advanced lines were derived from the two original inbreds, gradually increasing the superiority of the resulting hybrids.

A key difference between the cassava (KU50) and maize (Mo17xB73) hybrids is that KU50 cannot be re-made because its progenitors (R1 and R90) are not inbred. There are ways, however, to approximately define the respective haplotypes from R1 and R90 that produced the KU50 hybrid. Since R1 and R90 are still available, it is theoretically feasible to use reverse genetic approaches (Dirks et al. 2009; Guan et al. 2015; Wijnker et al. 2012) to produce partially or fully inbred lines that closely resemble the targeted haplotypes that defined KU50. This is a complex proposal, but once these inbred lines have been derived, a KU50 version, combining resistance to CMD and CBSD, could be developed and deployed in Asia and Africa. Having access to the inbred components of a strong heterotic pattern could be the basis for powerful alternative breeding approaches.

### Improving seed systems

A major challenge of cassava breeding programs compared with private seed businesses is that the former historically lack efficient systems to produce and distribute planting material of the new varieties. This delays the uptake and penetration of new varieties at the farm level, regardless of their genetic merit. While rapid propagation systems have been available, and continually refined for many years, practical, commercially viable systems have been nearly non-existent. There are ongoing efforts, primarily supported by the Bill and Melinda Gates Foundation in West Africa, to develop rapid multiplication approaches to produce disease-free planting materials. A goal of these systems is to make them sustainable as for-profit ventures.

### Breeding system decision-making and execution of research processes

To maximize genetic gains and the economic impact of cassava breeding programs, decision-making should arise from a consensus among the different actors of the team and be driven by client interests. An interdisciplinary cassava research team is required to determine the market segments and product profiles and develop and implement the Stage&Gate system. Cobb et al. (2019) present a perceived dichotomy between “*the -omics and machine learning and artificial intelligence potential of transforming plant breeding programs toward a data-rich, evidence-based, and team-oriented process and* away *from the romantic tradition of an individual breeder as an artist*.” This dichotomy, however, is fallacious. Plant breeders, by default, use best evidence in their decision-making; they are comfortable handling large databases and they typically work in teams. As an example of a field breeder searching for evidence-based proof of the advantages of a breeding approach, a large database (> 30,000 plots) was used to assess the value of breeding value in cassava (Ceballos et al. 2016a). It is also an example of reversing an earlier decision based on the evidence obtained from available data (Ceballos et al. 2004). Applied breeders, by nature, take a long-term, integrated view of challenges and opportunities, and adopt best-bet methods and tools. They usually avoid technology-based biases, their ultimate objective being to maximize genetic gain, impact at field level, and incorporation of the tools and expertise of the molecular breeder or gene-editing specialists, without bias for or against any particular class of tools. There is a key principle that is widely accepted: without high-quality phenotypic data, molecular tools are not useful. Quality of phenotypic data does not only include proper field management, but also careful management of planting material and reliable tracking of samples and data.

Major challenges that public cassava breeding programs face, from our perspective, are given as follows:

- Donors increasingly define the research agenda (Pingali and Kelley 2007).
- Novel breeding tools have become an end by themselves and their actual impact on improving breeding efficiency has not been properly assessed.
- Breeders and research managers need to work more closely together in order to assure that assignment of resources is based on the full range of complex goals of a breeding program.
- Innovation cannot become an end by itself, where new technologies are seen as better than old ones, irrespective of their actual impact. In the case of cassava, biotechnological approaches have been slower to yield results and tangible impact than many proponents promised.

Finally, we want to highlight an example of efforts to reach an integration between different actors in a cassava breeding program: the NextGen Project, managed by Cornell University. Many different collaborators and areas of expertise have been integrated toward a common goal (Mbanjo et al. 2021). Upstream genomic approaches have been combined with conventional and participatory breeding approaches. End-user demands have been incorporated into the definition of breeding objectives. Finally, key biological bottlenecks such as those related to flowering biology have been addressed and successfully solved (Hyde et al. 2019; Pineda et al. 2020a, 2020b). These principles and approaches epitomize the future of successful cassava breeding.

## Data Availability

This manuscript does not have additional associated data.
